# Overexpression of TMEFF1 in Endometrial Carcinoma and the Mechanism Underlying its Promotion of Malignant Behavior in Cancer Cells

**DOI:** 10.7150/jca.58524

**Published:** 2021-07-31

**Authors:** Xin Nie, Lingling Gao, Mingjun Zheng, Caixia Wang, Shuang Wang, Xiao Li, Yue Qi, Liancheng Zhu, Juanjuan Liu, Bei Lin

**Affiliations:** 1 Shengjing Hospital of China Medical University, Department of Obstetrics and Gynecology, Shenyang, China.; 2 Key Laboratory of Maternal-Fetal Medicine of Liaoning Province, Key Laboratory of Obstetrics and Gynecology of Higher Education of Liaoning Province, Shenyang, China.; 3 University Hospital, LMU Munich, Department of Obstetrics and Gynecology, Munich, Germany.; 4 West China Second University Hospital, Sichuan University, Department of Obstetrics and Gynecology, Sichuan, China.

**Keywords:** TMEFF1, endometrial carcinoma, EMT, MAPK, metastasis

## Abstract

**Background:** Although tomoregulin-1 (TMEFF1) is involved in embryonic development and central nervous system regulation and is a cancer suppressor gene in brain cancers, its role in endometrial carcinoma remains unclear.

**Methods:** The expression and prognostic value of TMEFF1 were analyzed by bioinformatics methods and immunohistochemistry. An endometrial carcinoma cell line with low expression of TMEFF1 was constructed. Scratch and Transwell assays were used to determine the effect of TMEFF1 on cell invasion and migration. Changes in key proteins in the MAPK and PI3K/AKT signaling pathways and in epithelial-mesenchymal transition (EMT)-related proteins were analyzed using western blot. Chromatin immunoprecipitation assay (ChIP) was performed to identify whether the TMEFF1 promoter region binds to the transcription factor p53.

**Results:** TMEFF1 was significantly upregulated in endometrial carcinoma, was closely associated with FIGO stage (*P*=0.021) and lymph node metastasis (*P*=0.029), and was an independent risk factor for prognosis (*P*=0.044). Gene Ontology (GO) and Kyoto Encyclopedia of Genes and Genomes (KEGG) pathway analyses showed that TMEFF1 and its related genes are involved in the cell cycle, regulation of mitosis, epigenetics, neural development, cell biological signal transduction and some key signal pathways. We also identified kinases, microRNAs and a transcription factor network related to TMEFF1 and the effect of TMEFF1 mutation on prognosis. *In vitro* knockdown of TMEFF1 significantly inhibited cell invasion and migration. Knockdown of TMEFF1 inhibited Epithelial-mesenchymal transition (EMT) and activation of the MAPK and PI3K/AKT pathways. However, the transcription factor p53 was not found to regulate the TMEFF1 gene.

**Conclusion:** TMEFF1 plays an important role in endometrial carcinoma and may thus be a potential anticancer therapeutic target for endometrial carcinoma.

## Introduction

Endometrial carcinoma is one of the common malignant cancers of the female reproductive system. In developed countries, the mortality rate is second only to ovarian cancer, and its incidence is increasing year by year 1. Endometrial carcinoma is usually divided into two major clinical pathological types: type I (estrogen-dependent) endometrial carcinoma, accounting for 80% of endometrial carcinomas; and type II (nonestrogen-dependent) endometrial carcinoma, including serous cancer and clear cell carcinoma, which usually occurs in the atrophic endometrium after menopause; type II has worse prognosis than type I 2. There are few methods for clinically treating endometrial carcinoma. In addition, the lesions cannot be completely removed in late-stage patients and the long-term recurrence rate is high. Moreover, the sensitivity of radiotherapy and chemotherapy is poor, with considerable toxicity, multiple adverse effects, and poor efficacy. Therefore, it is vital to identify new preventive methods 3.

Cancer/testis antigens (CTAs) are cancer-associated antigens that are mainly expressed in cells of the testis and embryonic tissues and lowly or not expressed in other normal tissues 4, 5. Because these antigens are expressed in different types of malignant cells, they are an antigenic target for the early detection of cancers and efficacy of cancer immunotherapy 6. TMEFF1 is the 120^th^ member of the CTA family 7. In early studies, TMEFF1 was a cancer suppressor in brain cancers 8, 9. Work showed that TMEFF1 was highly expressed in ovarian cancer tissues, promoted the malignant behavior of cancers, and was closely associated with the occurrence and progression of malignant cancers 10. A number of questions remain, such as the expression of TMEFF1 in endometrial carcinoma and whether it plays a role in suppressing or promoting cancer. There has been no research into TMEFF1 in endometrial carcinoma. Accordingly, we use several bioinformatics databases to analyze the expression of TMEFF1 in endometrial carcinoma and functional regulatory networks. Subsequently, we examined the expression of TMEFF1 in endometrial clinical samples and analyzed its clinical significance. We additionally investigated the effect of TMEFF1 on the malignant behavior of endometrial carcinoma and its mechanism, providing a theoretical basis for the early diagnosis and immunotherapy of endometrial carcinoma.

## Methods

### Oncomine database analysis

The Oncomine database (http://www.oncomine.org) 11 is an oncogene database and integrated data mining platform that enables analysis of differential expression in clinically common cancer tissues and their corresponding normal tissues.

### UALCAN analysis

UALCAN (http://ualcan.path.uab.edu) 12 is an effective online analysis tool and cancer database that enables the user to analyze data according to cancer stage, tumor grade, and patient race and weight, as well as the relative expression of genes of interest and clinicopathological characteristics in tumor and normal samples. It can also be used to estimate the impact of gene expression levels and clinicopathological characteristics on patient survival.

### LinkedOmics analysis

The LinkedOmics database (http://www.linkedomics.org/login.php) 13, 14 is an online platform for analyzing multiomics data and clinical data. The LinkFinder module was used to study the differentially expressed genes related to TMEFF1 in the TCGA uterine corpus endometrial carcinoma (UCEC) dataset, and Pearson correlation coefficients were determined and used to statistically analyze the results. Volcano plots, heatmaps and scatter plots were constructed for each differentially expressed gene related to TMEFF1. The LinkInterpreter module first standardizes and sorts all differentially expressed genes and then enriches and analyzes the signal pathways and network regulation of the genes of interest. The cutoff used was FDR <0.05, and 500 simulations were performed.

### Metascape analysis

Metascape (http://metascape.org)^15^ is a tool for gene annotation and analysis that integrates multiple authoritative database resources, such as the GO project, KEGG, UniProt and DrugBank. In this study, Metascape was used to analyze the enrichment of TMEFF1 and its related differentially expressed genes in processes and pathways. Gene Ontology (GO) biological analysis provides results in terms of three categories: biological process, cellular component, and molecular function. The following were used as cutoff values: *P* < 0.01, minimum count of 3, and enrichment factor >1.5.

### GeneMANIA analysis

GeneMANIA (http://www.genemania.org) 16 is an online platform that can predict gene function, analyze gene lists, and rank genes with clear functions. It is also used to construct protein-protein interaction (PPI) networks, protein-DNA interaction networks, and signaling pathways, determine gene and protein expression, and predict protein interaction domains and phenotypes, physiological and biochemical reactions and other processes. We used GeneMANIA to determine the proteins that interact with TMEFF1.

### STRING analysis

The STRING database (https://string-db.org/) 17 is a database for searching for interactions between proteins, including both direct physical interactions between proteins and indirect functional correlations between proteins. We used the STRING database to analyze the proteins related to TMEFF1.

### GEPIA analysis

The GEPIA database (http://gepia.cancer-pku./cn/index.html) 18 is an interactive web server used to integrate and analyze cancer expression profile data, including RNA sequencing expression data of tumor and normal samples from TCGA and GTEx. We used the “General” module of this online analysis tool to analyze the expression level of the TMEFF1 gene in various tumor tissues, and the “Expression DIY” module was used to analyze the expression of the TMEFF1 gene in UCEC. The screening conditions used in the “Expression DIY” module are as follows: gene: TMEFF1; dataset: UCEC; log2FC cutoff > 1; and *P* < 0.01.

### cBioPortal analysis

The data from the cBioPortal database (https://www.cbioportal.org/) 19 come from TCGA, ICGC, GEO and other databases. The types of integrated genome data include somatic mutation, DNA copy number alteration (CNA), mRNA and miRNA expression, DNA methylation, protein abundance and phosphoprotein abundance data. cBioPortal can visualize genomic data of cancer research samples and genetic differences between samples as well as genes and pathways and can compare them with clinical results.

### Specimen source and clinical data

Surgical paraffin-embedded pathology specimens were collected from 135 patients at the Department of Obstetrics and Gynecology, Shengjing Hospital affiliated to China Medical University, from 2004 to 2017. The pathological diagnosis of all histological sections was completed by a pathologist of Shengjing Hospital affiliated to China Medical University. There were 75 cases of endometrial carcinoma, 24 cases of endometrial atypical hyperplasia (8 of mild hyperplasia, 9 of moderate hyperplasia, and 8 of severe hyperplasia), and 36 cases of normal endometrium (21 in the secretory phase and 15 in the proliferative phase). Normal endometrial tissue was obtained from patients who had undergone total hysterectomy and bilateral adnexectomy or total hysterectomy due to cervical lesions, without fertility requirements. In addition, the atypical hyperplasia and normal endometrial tissue samples were from patients without uterine fibroids, chocolate cyst of the ovary, or other estrogen-dependent diseases.

The patients in the endometrial carcinoma group were 36 to 79 years old, with an average age of 60.0 years; the patients in the endometrial atypical hyperplasia group were 35 to 66 years old, with an average age of 47.5 years; and the patients in the normal endometrium group were 39 to 53 years old, with an average age of 44.1 years. There was no significant difference in the ages of each group (*P*>0.05). The pathological types of endometrial carcinoma were as follows: 37 cases of endometrioid adenocarcinoma, 23 cases of serous papillary adenocarcinoma, 8 cases of clear cell carcinoma, and 7 cases of other pathological types (3 of mucinous carcinoma, 2 of squamous-cell carcinoma, and 2 of undifferentiated carcinoma). According to the pathological grades, 16 cases were highly differentiated, 29 were moderately differentiated, and 30 were poorly differentiated. According to the International Federation of Gynecology and Obstetrics (FIGO) 2009 staging, 50 cases were FIGO stage I, 6 were stage II, 16 were stage III, and 3 were stage IV. There were 14 cases of lymph node metastasis, 48 without lymph node metastasis, and 13 without lymph node dissection. There were 48 cases with < 1/2 muscle infiltration and 27 with ≥ 1/2 muscle infiltration. All patients had primary endometrial carcinoma, the clinical and pathological data were complete, and no radiotherapy or chemotherapy was performed before the surgery. Nine of the patients did not undergo estrogen receptor (ER) and progesterone receptor (PR) tests. The study was approved by the Ethics Committee of China Medical University. Written informed consent was obtained from all participants.

### Immunohistochemistry

Endometrial tissue was serially sectioned at 5 μm. The expression of TMEFF1 was determined using the immunohistochemical streptavidin-peroxidase ligation (SP) method. The staining method was carried out according to the SP kit manual (China Maxim). The positive control used normal human epididymal tissue and the negative control replaced the primary antibody with homologous IgG. The TMEFF1 antibody concentration was 1:70 (Bioss, China). Brown particles were evident in the cell membrane and/or cytoplasm and were classified according to color intensity as follows: 0 points, uncolored; 1, light yellow; 2, brown; and 3, dark brown. Positive cell rates < 5%, 6-25%, 26-50%, 51-75%, and > 75% were scored as 0, 1, 2, 3, and 4, respectively. The final score was obtained by multiplying the score of the two items mentioned above: 0-2 was considered negative (-), 3-4 was (+), 5-8 was (++), and 9-12 was (+++). Two observers participated in the scoring to control for errors.

### Cell culture and TMEFF1 gene transfection

The endometrial carcinoma cell lines Ishikawa and HEC1B were purchased from the Shanghai Cell Bank of the Chinese Academy of Sciences. The cells were routinely cultured in McCoy's 5A/DMEM medium containing 10% fetal calf serum (Gibco, USA) at 37 °C, 5% CO_2_, and saturated humidity. Using siRNA transfection, cell lines with low expression of TMEFF1 and negative control cell lines were established and named Ishikawa-TMEFF1-siRNA, Ishikawa-NC, HEC-1-B-TMEFF1-siRNA and HEC-1-B-NC. The blank control was named Ishikawa-Blank and HEC-1-B-Blank. Two siRNAs showed synergic effects on the knockdown of TMEFF1. The TMEFF1 siRNA sequence 1 (Genepharma, China) was as follows: sense, 5'-GCUCACUCAUGUUCUUAUUTT-3'; antisense: 5'- AAUAAGAACAUGAGUGAGCTT-3'. The TMEFF1 siRNA sequence 2 (Genepharma, China) was as follows: sense, 5'-TGCTGACTAAAGTCCGTCTTCTCACAG-3'; antisense: 5'- TTTTGGCCACTGACTGACTGTGAGAACGGACTTTAGT-3'.

### Real-time quantitative PCR

Total mRNA was extracted from endometrial carcinoma cells using TRIzol (TAKARA, Japan). The cDNA was then synthesized by reverse transcription of RNA using the Super Script III First-Strand Synthesis System RT-PCR kit (TAKARA). The real-time quantification procedure was performed via a two-step method: pre-denaturation at 95 °C for 30 s, followed by denaturation at 95 °C for 5 s and annealing at 60 °C for 31 s for a total of 40 cycles. With GAPDH as an internal reference, real-time PCR amplification was performed using an AB17500 PCR machine to detect the Ct value of each template. After the amplification, a dissolution curve analysis was performed. The fold-changes were calculated using the 2^-ΔΔCt^ method. The experiment was repeated three times. The TMEFF1 primer (Sangon, Shanghai, China) was as follows: forward, 5'-TTGTTGGGAA AGAAAGATGA TGGA-3'; reverse, 5'-GATGCAGTAA CCATTGAGGT TTT-3'.

### Western blot

Total protein was extracted from endometrial carcinoma cells and denatured. The protein was separated by 10% sodium dodecyl sulfate-polyacrylamide gel electrophoresis (SDS-PAGE) and transferred to a methanol-activated PVDF membrane. After blocking for 1 h using 5% milk or BSA, the PVDF membrane was incubated with primary antibody at 4 °C overnight. The primary antibodies were as follows: anti-TMEFF1 antibody (Santa Cruz, 1:500); anti-PI3K p85 antibody (Cell Signaling Technology, 1:1000), anti-phospho-PI3K p85 antibody (Ser458) (Cell Signaling Technology, 1:1000), anti-AKT antibody (Cell Signaling Technology, 1:1000), anti-phospho-AKT (Ser473) antibody (Cell Signaling Technology, 1:1000), anti-MEK1/2 antibody (Cell Signaling Technology, 1:1000), anti-phospho-MEK1/2 antibody (Cell Signaling Technology, 1:1000), anti-ERK1/2 antibody (Cell Signaling Technology, 1:1000), anti-phospho-ERK1/2 antibody (Cell Signaling Technology, 1:1000), anti-E-cadherin antibody (Cell Signaling Technology, 1:1000), anti-Vimentin antibody (Proteintech, 1:1000), anti-MMP2 antibody (Proteintech, 1:1000), anti-MMP9 antibody (Proteintech, 1:1000), and anti-GAPDH (Zhongsu Jinqiao, 1:2000). After washing with TBST, the membrane was incubated with secondary antibody (Nakasugi Jinqiao, 1:5000) for 2 h at room temperature. Protein bands were measured by ImageJ 1.31v and normalized to the expression levels of GAPDH.

### Scratch test

Cells were seeded in a 6-well plate. After the cell density reached 90%, the plate was gently scratched using a 100-µl pipette tip. The cells were then cultured in serum-free medium for 0 and 24 h and the width of the scratch was observed under a microscope. The experiment was repeated three times.

### Transwell assay

Matrigel gel (80 µl) was added to the upper chamber of a Transwell chamber (Corning Costar, USA) in a 37 °C incubator for 5 h. Then, 500 μl of 10% fetal bovine serum culture medium was added to the lower chamber, and 200 μl of a cell suspension in serum-free medium (2 × 10^5^ cells) was added to the upper chamber. The cells were incubated at 37 °C for 48 h. The upper chamber surface was gently wiped with a cotton swab and the cells in the lower chamber were fixed in 4% paraformaldehyde at room temperature for 25 min and stained with crystal violet for 25 min. The numbers of cancer cells that infiltrated the filter membrane were counted under a microscope. Three replicate wells were set per well and the experiment was repeated three times.

### Chromatin immunoprecipitation (ChIP) assay

Endometrial cancer cells (ISHIKAWA cells) were collected during the logarithmic growth phase. A commercially available kit (CST, 9004) and p53 antibody (CST, 2527s) were used for ChIP analysis, which was carried out according to the manufacturer's instructions. Real-time PCR was performed with the following primers: forward primer F covering the promoter region of TMEFF1: 5'-ATGGCTAGAGTCAGAACTTG-3'; reverse primer R: 5'-TGAGTCACGGAAGAGGTAA-3'. The negative control sequence was amplified by a primer that binds to the promoter region of the HCII gene, not within the TMEFF1 promoter or any other part of TMEFF1: forward primer F of the negative control: 5'-TTATGTGGTGACCTCAAGAG-3'; reverse primer R: 5'-TGACGGTTACTGTGTTAGC-3'. The experiment was repeated three times.

### Statistical analysis

The study data were analyzed with SPSS22.0 software. The measurement data were analyzed by single-factor analysis of variance. The counting data were analyzed by *x^2^* test and Fisher exact probability test. The survival curve was analyzed by Kaplan-Meier and log-rank tests. The relationship with patients was analyzed using Cox model. *P* < 0.05 indicated that the difference was statistically significant.

## Results

### Analysis of TMEFF1 expression and prognosis in the Oncomine database, GEPIA database and UALCAN

**Figure 1:**
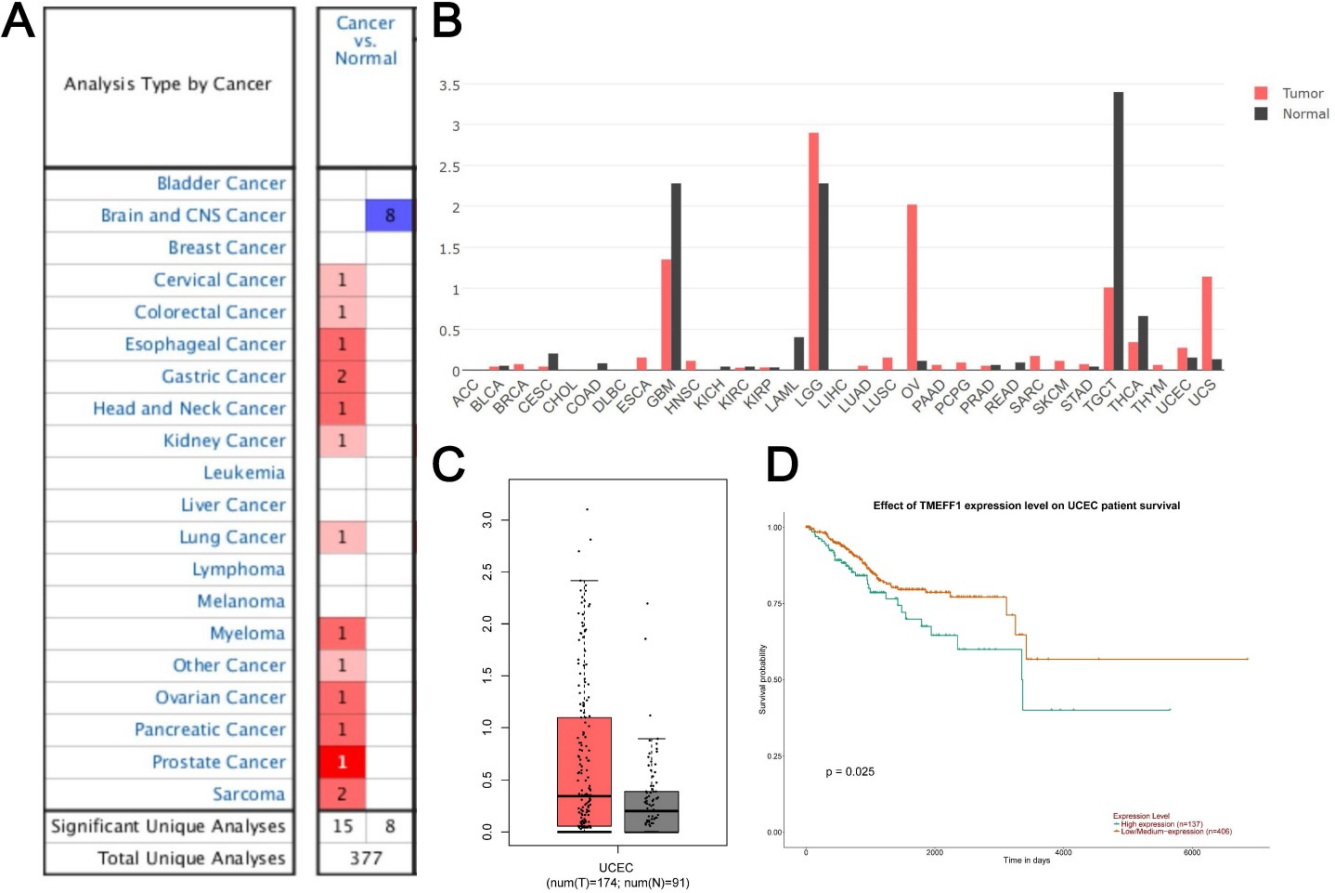
** The expression of TMEFF1 in the Oncomine dataset and GEPIA dataset of endometrial cancer patients.** (**A**) TMEFF1 mRNA levels in different types of tumors in the Oncomine database. (**B**) TMEFF1 mRNA levels in different types of tumors in the GEPIA database. (**C**) TMEFF1 mRNA levels in endometrial cancer in the GEPIA database. (**D**) Correlation analysis of TMEFF1 expression with overall survival in UALCAN. Data are presented as the mean ± SE.

Data on TMEFF1 expression from studies in 377 different types of tumors were extracted from the Oncomine database. 23 studies showed significant differences in TMEFF1 mRNA levels. Among them, there were 15 studies with significantly higher expression of TMEFF1 mRNA and 8 studies with significantly lower expression of TMEFF1 mRNA. The expression of TMEFF1 mRNA was significantly increased in cervical cancer, colorectal cancer, esophageal cancer, gastric cancer, head and neck cancer, kidney cancer, lung cancer, myeloma, ovarian cancer, pancreatic cancer, prostate cancer and sarcoma but decreased in brain and CNS cancer (Fig. 1A). GEPIA website analysis showed that the expression of TMEFF1 mRNA was significantly increased in ovarian cancer, uterine corpus endometrial carcinoma, and uterine carcinosarcoma, but decreased in glioblastoma multiforme, acute myeloid leukemia, and thyroid carcinoma (Fig. 1B). The analysis of TMEFF1 in 174 endometrial cancer tissues and 91 normal endometrial tissues indicated that TMEFF1 mRNA was significantly highly expressed in endometrial cancer (Fig. 1C) (*P* < 0.01). Further analysis using the UALCAN website found that compared with patients with low/medium TMEFF1 expression (406 patients), the survival time of patients with high TMEFF1 expression (137 patients) with endometrial cancer was significantly shorter (P=0.025) (Fig. 1D), suggesting that patients with endometrial cancer with high TMEFF1 expression have a poor prognosis.

### TMEFF1 expression in endometrial tissue

#### TMEFF1 is highly expressed in endometrial carcinoma

**Figure 2:**
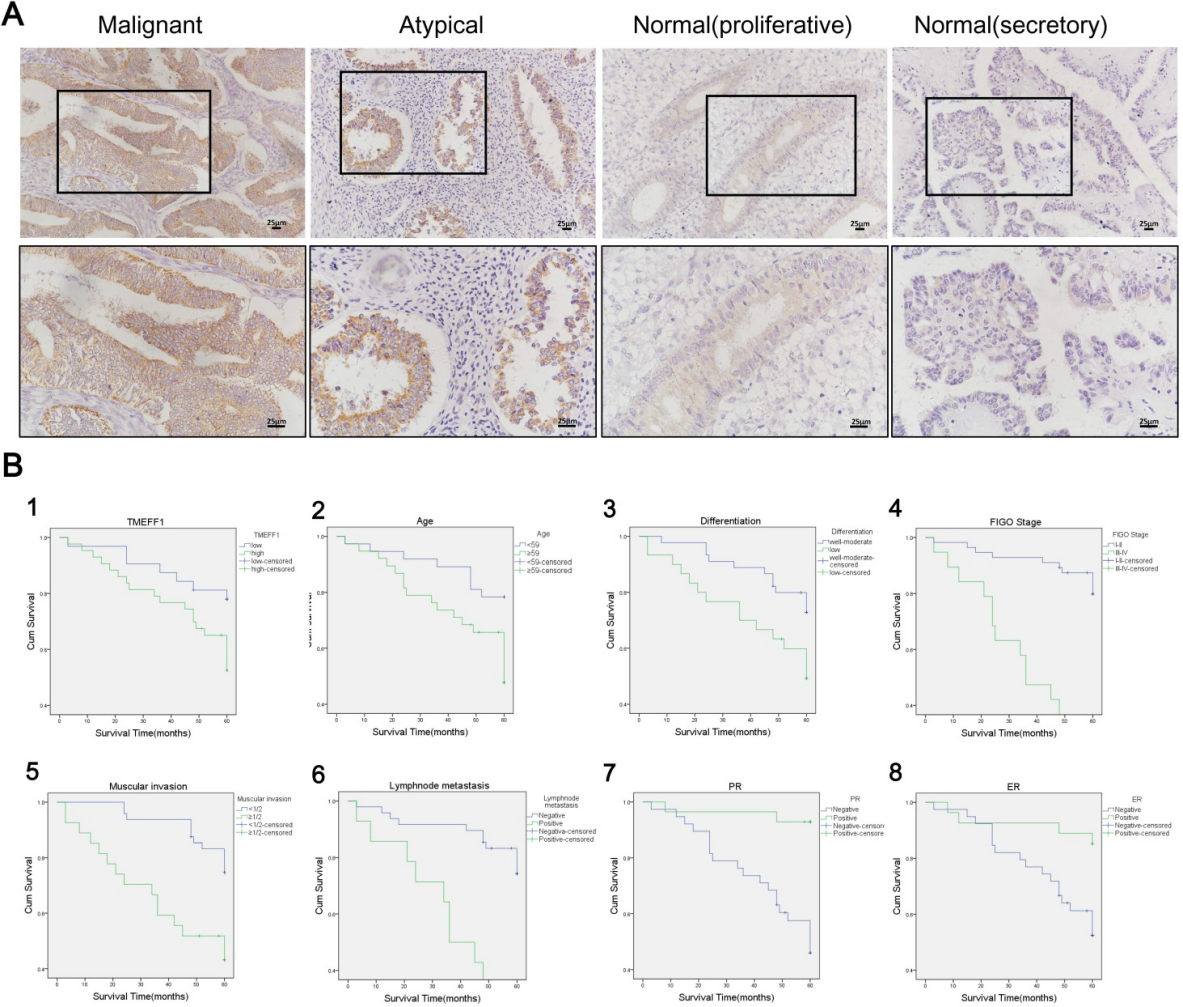
** TMEFF1 is significantly upregulated in various endometrial carcinomas and is significantly associated with patient prognosis.** (**A**) Expression of TMEFF1 in various endometrial tissues (×200; ×400). (**B**) Analysis of factors related to endometrial prognosis. 1: TMEFF1 expression level; 2: age at diagnosis; 3: degree of differentiation; 4: FIGO stage; 5: depth of myometrial invasion; 6: lymphatic metastasis; g: PR(+); h: ER(+).

TMEFF1 was mainly stained at the membrane, although staining was also evident in the cytoplasm (Fig. 2A). The positive expression rate of TMEFF1 in endometrial carcinoma was 82.67% (62 of 75 cases), which was significantly higher than that of atypical hyperplasia (62.50% [15 of 24]) and normal endometrial tissue (58.33% [21 of 36]) (*P*=0.039 and 0.006, respectively). The high expression rate of TMEFF1 in endometrial carcinoma was 57.33% (43 of 75), which was significantly higher than that of normal endometrial tissue (25.00% [9 of 36]) (*P*=0.001). The positive expression rates of TMEFF1 in severe, moderate, and mild endometrial atypical hyperplasia were 85.71%, 55.56%, and 50.00%, respectively, with the expression increasing with the severity of the lesion, although the difference was not statistically significant (*P*>0.05). The positive rate of secretory endometrial tissue was 61.90% (13 of 21), that of proliferative endometrial tissue is 53.33% (8 of 15), and the difference was not statistically significant (*P*>0.05) (Fig. 2A and Table 1).

#### Relationship between TMEFF1 expression and clinicopathological parameters in endometrial carcinoma

This study included 75 cases of endometrial carcinoma. The positive rate and high expression rate of TMEFF1 in early-stage cancer (I-II) were 76.78% (43 of 56) and 50.00% (28 of 56), respectively, which was significantly lower than those in the late stage (III-IV) (100.00% [19 of 19] and 78.95% [15 of 19]; *P*=0.021 and 0.027, respectively). The positive expression rate of TMEFF1 in the positive lymph node metastasis group was 100% (14 of 14), which was significantly higher than that in the negative lymph node metastasis group (72.92% [35 of 48]) (*P*=0.029). The expression of TMEFF1 was not significantly associated with pathological type, degree of differentiation, PR(+) and ER(+) status, or depth of myometrial invasion (Table 2).

#### Survival analysis and risk factors for prognosis of endometrial carcinoma

Until November 2018, the patients' survival time ranged from 3 to 116 months. In total, 23 patients with endometrial carcinoma died due to cancer recurrence, 4 patients survived after cancer recurrence, 43 patients had cancer-free survival, 1 patient had non-recurrent death, and 4 patients were lost to follow-up. Univariate Kaplan-Meier analysis and log-rank test showed that high TMEFF1 expression was significantly associated with worse overall survival (*P*=0.035). The high TMEFF1 expression group had a median survival time of 43 months, whereas the survival time in the low TMEFF1 expression group was 54 months. In addition, age at diagnosis (<59 years vs ≥59 years), FIGO stage (I-II vs III-IV), type of differentiation (medium-high differentiation vs poor differentiation), depth of myometrial invasion (<1/2 vs ≥1/2), lymph node metastasis (- vs +), and PR and ER status (- vs +) were also associated with poor prognosis (*P* < 0.05) (Fig. 2B and Table 3).

We used Cox regression analysis to explore the relationships among different clinicopathological parameters, TMEFF1 expression, and prognosis. In addition to the age of diagnosis, FIGO stage, degree of differentiation, depth of myometrial invasion, lymph node metastasis and PR and ER status, high expression of TMEFF1 also affected the overall survival of patients with endometrial carcinoma and was an independent risk factor for prognosis. The higher expression of TMEFF1, the worse the prognosis (*P*=0.044) (Table 4).

### Enrichment analysis of TMEFF1 functional networks in UCEC

**Figure 3:**
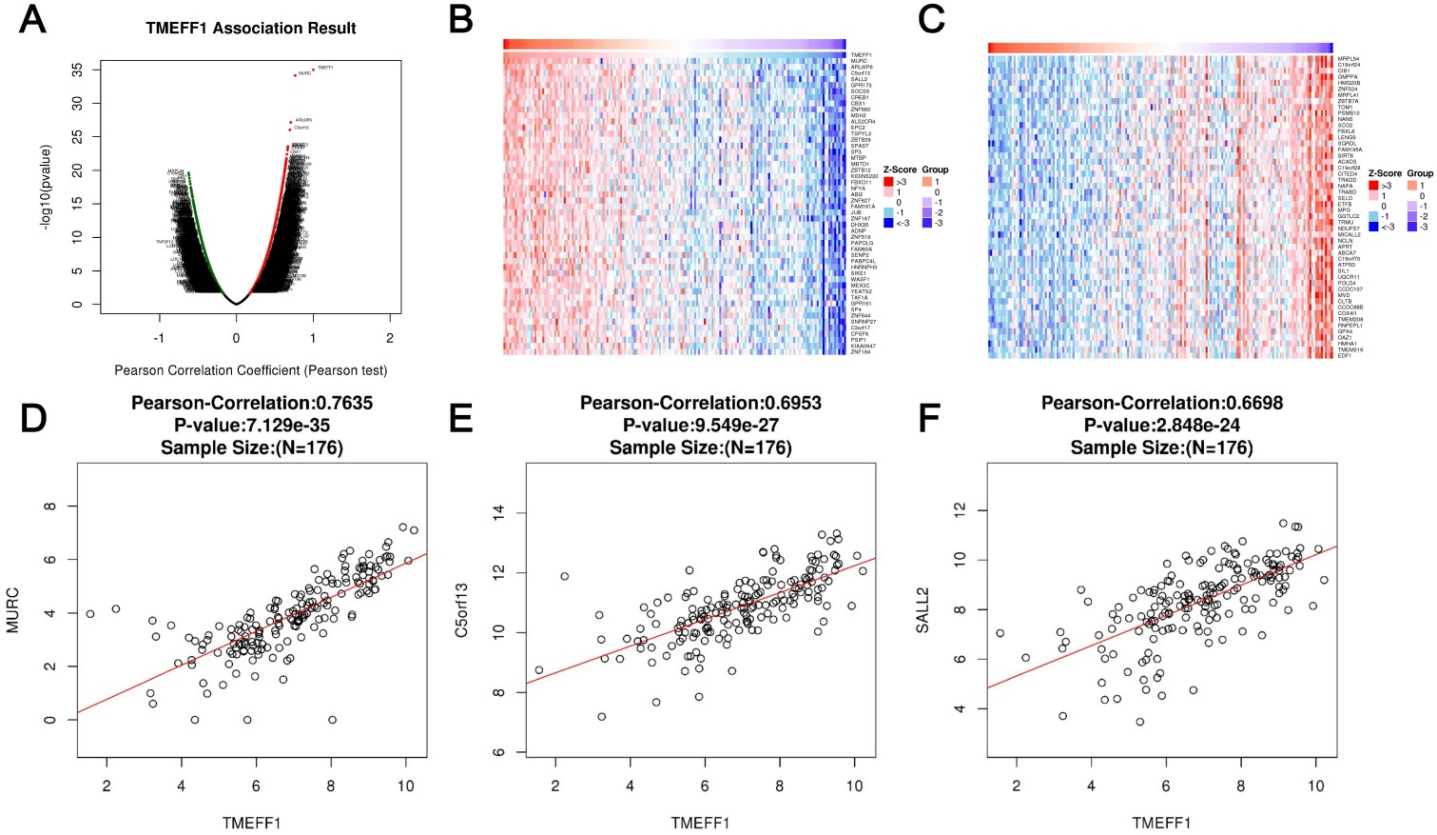
** Differentially expressed genes correlated with TMEFF1 in endometrial carcinoma.** (**A**) Pearson's test was used to analyze the correlation between TMEFF1 and differentially expressed genes in UCEC. (**B** and** C**) Genes positively correlated and negatively correlated with TMEFF1 in UCEC, as shown in heatmaps (top 50). Red indicates positively correlated genes, and green indicates negatively correlated genes. (**D-F**) Correlation of TMEFF1 expression with the expression of MURC (**D**), C5orf13 (**E**) and SALL23 (**F**) according to Pearson's test, as shown in scatter plots.

LinkedOmics was used to analyze the mRNA sequencing data of 176 UCEC patients in the TCGA database. As shown in the volcano map, there were 1640 genes that were significantly positively related to TMEFF1 (dark red dots) and 487 genes that are significantly negatively related to TMEFF1 (dark green dots) (false discovery rate [FDR] < 0.01). The heatmap shows the first 50 gene sets that are significantly positively and negatively correlated with TMEFF1 (Fig. 3A-C). The results show that TMEFF1 has a wide range of functions in regulating body metabolism, protein activity and information exchange between cells. The scatter plot of single genes shows that the expression of TMEFF1 was significantly positively correlated with the expression of MURC (positive rank #1, Pearson correlation=0.7635, P=7.129e-35), C5orf13 (Pearson correlation=0.6953, P=9.549e-27) and SALL2 (Pearson correlation=0.6698, P=2.848e-24) (Fig. 3D-F), and these genes play an important role in cardiomyocyte function, neuron regeneration and embryonic eye development.

**Figure 4:**
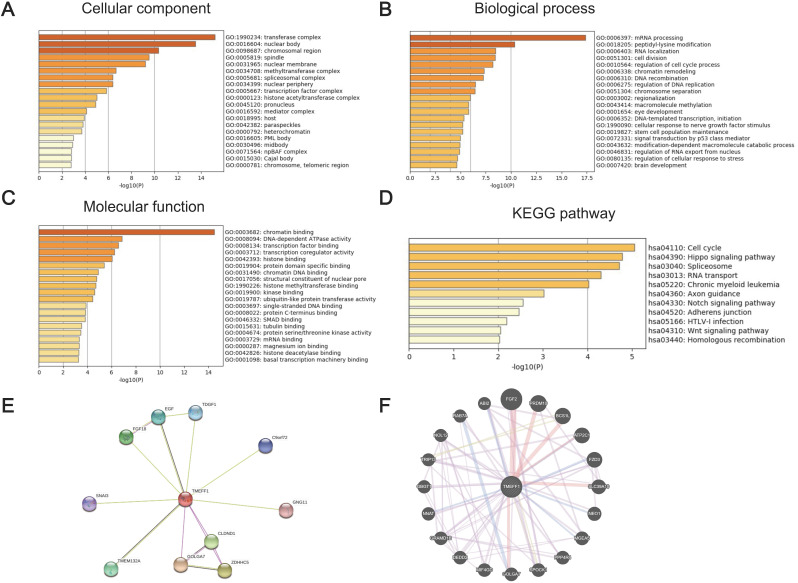
** The significantly enriched GO annotation and KEGG pathway terms of genes coexpressed with TMEFF1 in UCEC were analyzed with Metascape, and proteins interacting with TMEFF1 were analyzed in the STRING database and GeneMANIA database.** (**A**) Top 20 enriched with cellular component terms for the TMEFF1-related genes, as shown in a bar graph colored by *P*-values. (**B**) Top 20 enriched biological process terms for the TMEFF1 co-expressed genes, as shown in a bar graph colored by *P*-values. (**C**) Top 20 enriched molecular function terms for to the TMEFF1 co-expressed genes, as shown in a bar graph colored by *P*-value. (**D**) KEGG enriched terms colored by *P*-value. (**E**) PPI analysis using the STRING database. (**F**) PPI analysis using the GeneMANIA database.

GO and KEGG enrichment analyses were performed to determine the functions of TMEFF1 and related differentially expressed genes. The GO results showed that TMEFF1 and its associated genes are mainly located in transferase complexes, the nuclear body, the chromosomal region, the spindle, the nuclear membrane, the nuclear periphery, and chromosomes (Fig. 4A and Table S1). In addition, they are mainly involved in the cell cycle, the regulation of mitosis, epigenetics, neurodevelopment, cell biological signal transduction and other biological processes, such as mRNA processing, peptidyl-lysine modification, cell division, regulation of the cell cycle, chromatin remodeling, cellular response to nerve growth factor stimulus, brain development, signal transduction by p53 class mediators, etc. (Fig. 4B and Table S2). The molecular functions of TMEFF1 and its associated genes were found to mainly include regulating the activities of DNA-dependent ATPases, transcription coregulators, ubiquitin-like protein transferases, and protein serine/threonine kinases, and TMEFF1 and its associated proteins were suggested to be able to bind to chromatin, transcription factors, histones, protein domains, chromatin DNA, single-stranded DNA, protein C termini, tubulin, etc.; they can also interact with histone methyltransferases, kinases, histone deacetylase, etc. (Fig. 4C and Table S3).

The KEGG enrichment analysis results showed that the signaling pathways involving TMEFF1 and its associated genes include the cell cycle, Hippo signaling, spliceosome, RNA transport, and chronic myeloid leukemia pathways. (Fig. 4D and Table S4).

### TMEFF1-related kinase, miRNA or transcription factor target network in endometrial cancer

To further explore the functional targets of TMEFF1 in endometrial cancer, we analyzed the kinases, miRNAs and transcription factors that could serve as targets of genes positively related to TMEFF1 by GSEA. The top 5 identified kinases were ATM serine/threonine kinases, TTK protein kinases, ATR serine/threonine kinases, polo-like kinase 1, and cyclin-dependent kinase 2, which are mainly related to DNA damage, mitosis and the cell cycle. The miRNA target network included MIR-381, MIR-154, MIR-487, MIR-30A-3P, MIR-30E-3P, MIR-369-3P, MIR-212, and MIR-132. The transcription factor target network included proteins mainly related to DNA replication and repair and cell cycle regulation, including V$E2F1_Q6, V$E2F_02, V$E2F1DP1_01, V$E2F1DP2_01, and V$E2F4DP2_01 (Table 5 and Table S5-S7).

### PPI analysis using the STRING database and GeneMANIA database

To better understand the relationship between TMEFF1 and UCEC, we used STRING and GeneMANIA to perform protein-protein interaction (PPI) enrichment analysis (Fig. 4E and F). The results show that proteins that interact with TMEFF1 are involved in cell proliferation, differentiation, migration, apoptosis, autophagy, cell adhesion, protein transport, calcium transport, and various signal transduction processes and promote the development of nerves and embryos.

### Genomic changes in TMEFF1 in endometrial cancer

**Figure 5:**
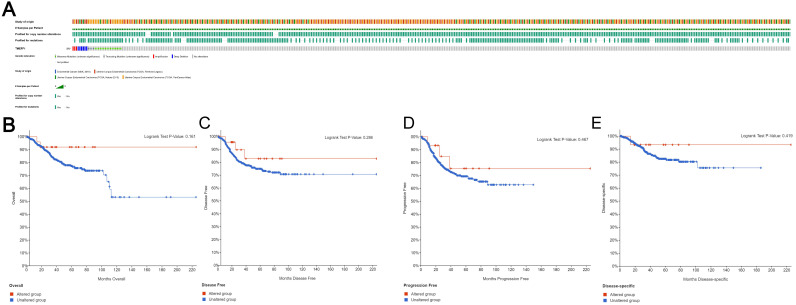
** TMEFF1 gene mutations and their effect on the survival and prognosis of endometrial carcinoma patients.** (**A**)Mutations of the TMEFF1 gene in the cBioPortal database. (**B-E**) Effect of the mutations of the TMEFF1 gene on the OS (**B**), DFS (**C**), PFS (**D**), and DSS (**E**) of endometrial carcinoma patients.

Based on the sequencing data of UCEC patients in the TCGA database, we used cBioPortal to determine the type and frequency of TMEFF1 changes in UCEC. TMEFF1 was altered in 29 of the 1638 (2%) UCEC patients (Fig. 5A). These alterations included the following: amplification (3 patients; 0.2%), deep deletion (6 patients; 0.4%), truncating mutation of unknown significance (4 patients; 0.2%) and missense mutation of unknown significance (16 patients; 1%). These results show that the TMEFF1 gene mutation has a very low incidence in endometrial cancer. And TMEFF1 gene mutation had no significant effect on Overall Survival (OS), Disease Free Survival(DFS), Progression Free Survival (PFS) or Disease Specific Survival(DSS) in patients with endometrial cancer (*P*>0.05) (Fig. 5B-E).

### Establishment of a low TMEFF1 expression endometrial carcinoma cell line

**Figure 6:**
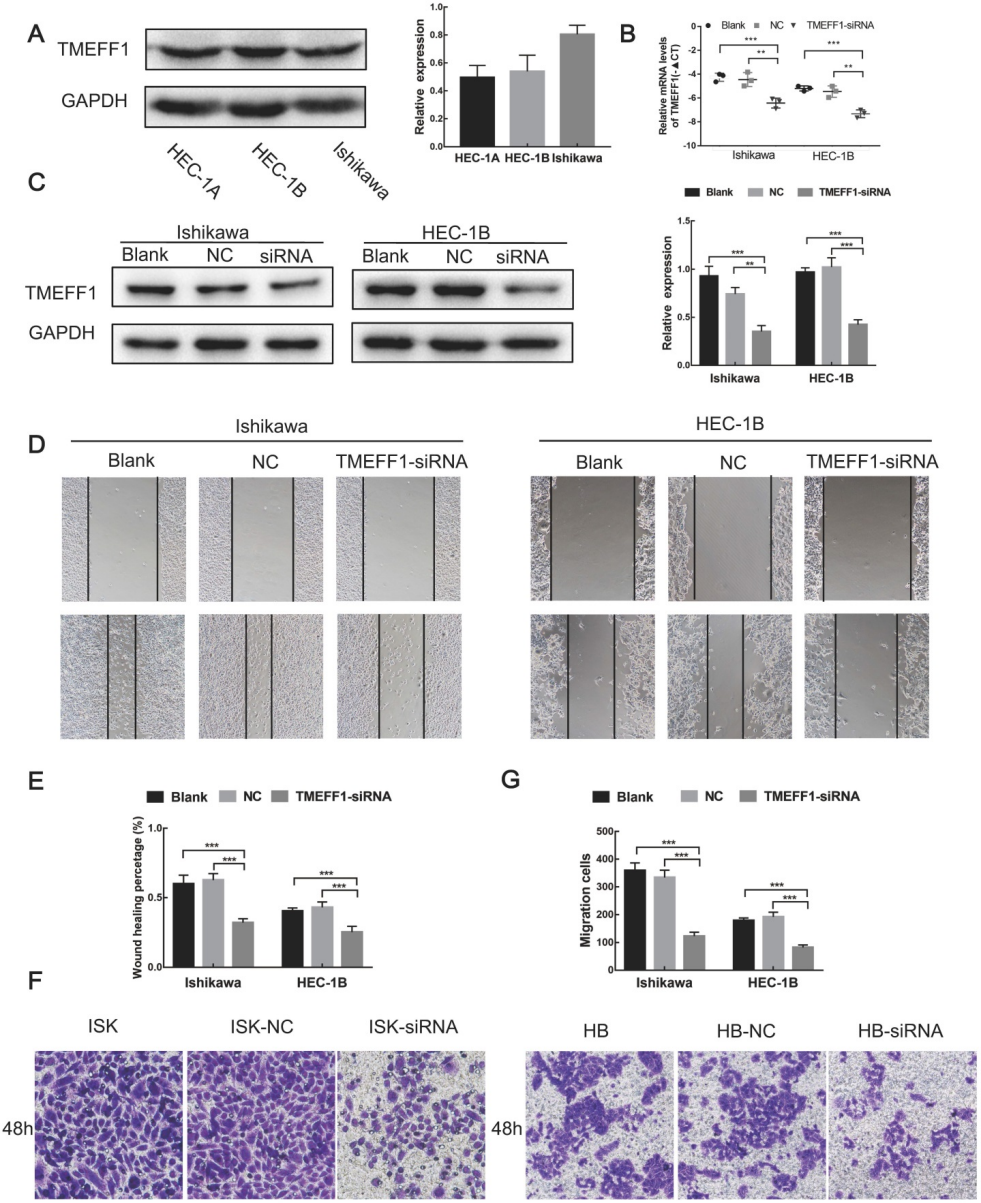
** Knockdown of TMEFF1 inhibits the migration and invasion of Ishikawa and HEC-1B cells.** (**A**) Expression of TMEFF1 in endometrial carcinoma cell lines. TMEFF1 (Molecular Weight): 41kDa; GAPDH (Molecular Weight): 36kDa. (**B** and** C**) After TMEFF1 knockdown in the intimal cancer cell lines Ishikawa and HEC-1B, the expression of TMEFF1 mRNA and protein was determined. (**D** and **E**) Scratch test to determine the effect of TMEFF1 expression on the migration of Ishikawa and HEC-1B cells. (**F** and **G**) Transwell assay assessing the effect of TMEFF1 expression on the invasion of Ishikawa and HEC-1B cells. Data are presented as the mean ± SEM (n = 3 per group).**P < 0.01 and ***P < 0.001.

We first compared the expression levels of TMEFF1 in different endometrial carcinoma cell lines (Ishikawa, HEC-1-B, and HEC-1-A) and then selected Ishikawa and HEC-1-B cell lines with high TMEFF1 expression for knockdown of the TMEFF1 gene. The interference efficiency was verified by real-time PCR and western blot experiments, with the expression of TMEFF1 gene and protein significantly lower in the interference group than in the control group (Fig. 6A-C).

### TMEFF1 knockdown inhibits the migration of endometrial carcinoma cells

Cell scratch experiments showed that the migration ability of Ishikawa and HEC-1-B cells decreased after knockdown of the TMEFF1 gene (Fig. 6D and E), indicating that TMEFF1 promotes the migration of endometrial carcinoma cells.

### TMEFF1 knockdown inhibits the invasion of endometrial carcinoma cells

Transwell assays revealed that TMEFF1 knockdown in Ishikawa and HEC-1-B cell lines significantly reduced cell invasiveness (Fig. 6F and G), indicating that TMEFF1 promotes the invasion of endometrial carcinoma cells.

### TMEFF1 affects key proteins involved in the regulation of EMT

**Figure 7:**
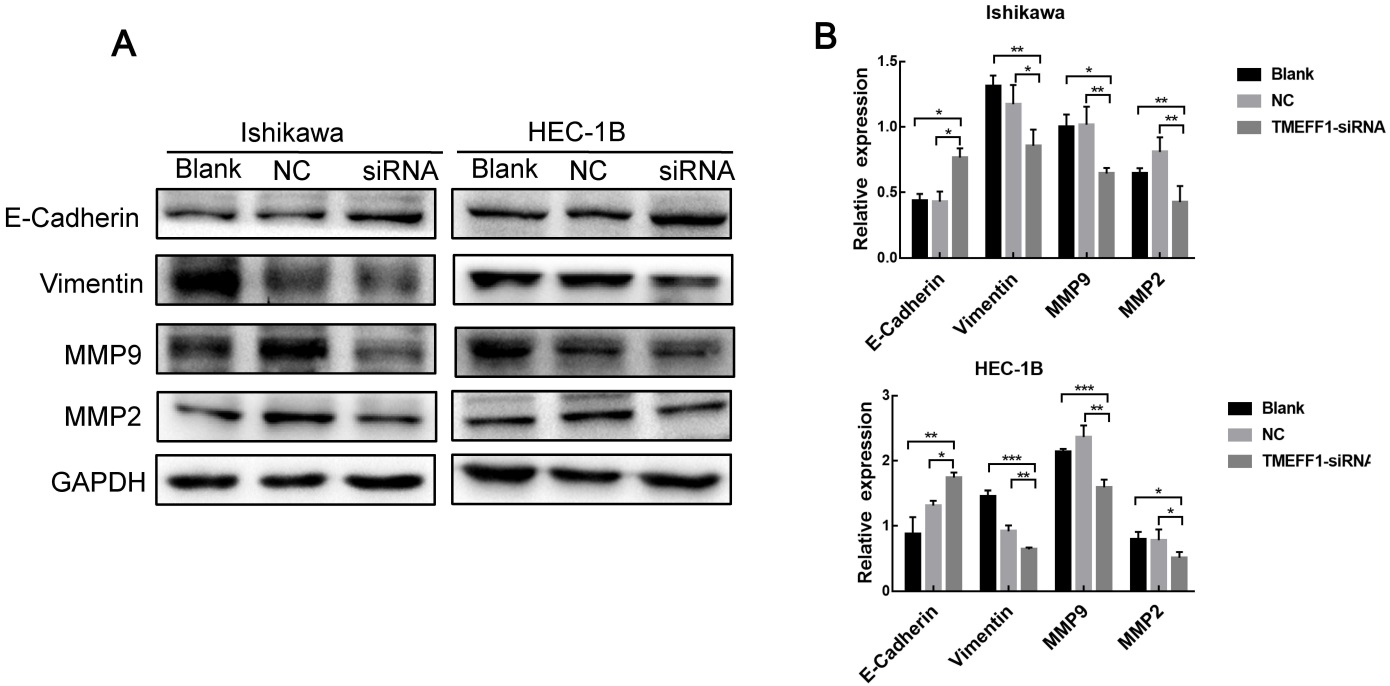
** TMEFF1 is involved in the promotion of epithelial-mesenchymal transition in Ishikawa and HEC-1B cells.** (**A** and **B**) Western blot analysis indicating that TMEFF1 increases the expression of vimentin, MMP2, and MMP9 in Ishikawa and HEC-1B cells and decreases the expression of E-cadherin. Data are presented as the mean ± SEM (n = 3 per group).*P < 0.05, **P < 0.01, and ***P < 0.001.

EMT plays an important role in the early stages of the metastatic spread of cancer by inducing cell movement and allowing cells to acquire invasive potential. To further explore the mechanism underlying the effect of TMEFF1 on the invasion and migration of endometrial carcinoma cells, we used western blot to determine the changes in the key EMT proteins E-cadherin, Vimentin, MMP2, and MMP9 between before and after TMEFF1 knockdown. The results showed that Vimentin, MMP2, and MMP9 expression levels were decreased and E-cadherin increased after inhibition of the expression of the TMEFF1 gene (Fig. 7A and B). Thus, TMEFF1 is involved in the regulation of EMT and affects the malignant behavior of endometrial carcinoma cells.

### TMEFF1 activates PI3K of the AKT and MAPK signaling pathways

**Figure 8:**
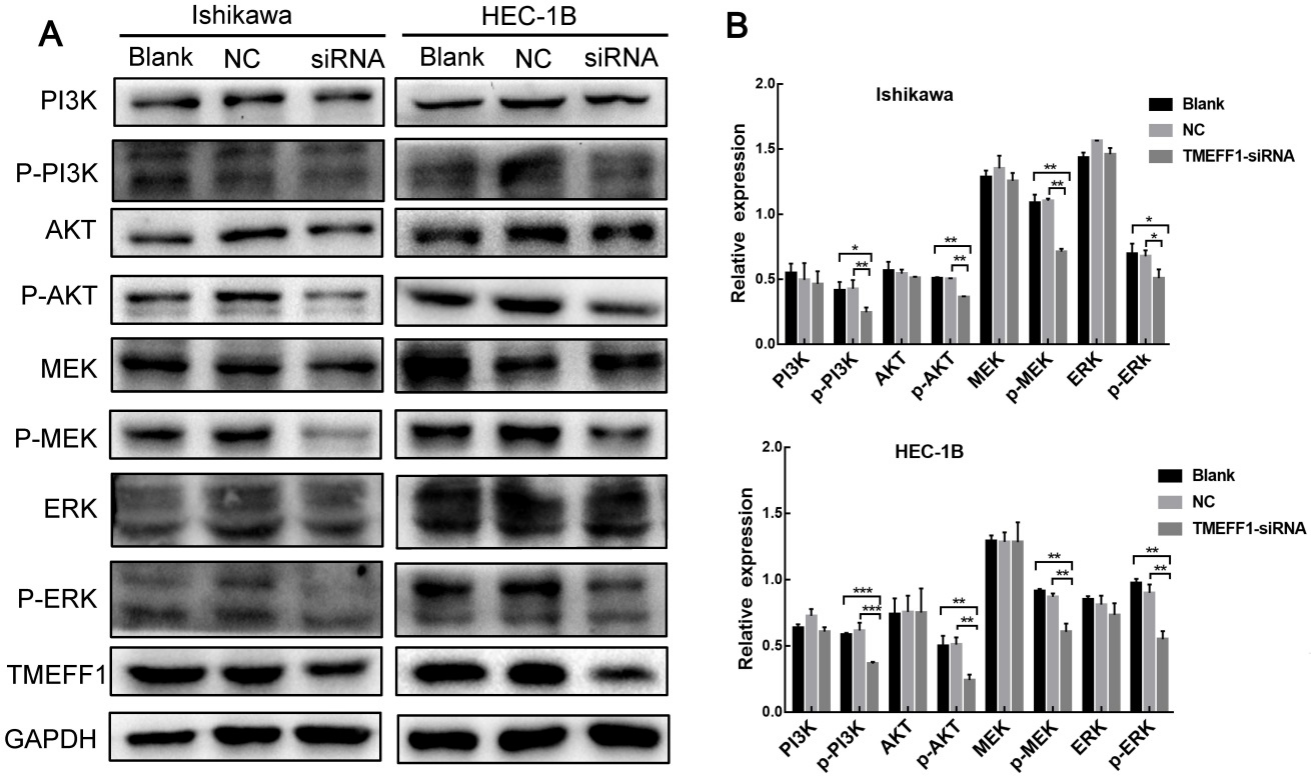
** TMEFF1 activates the MAPK and PI3K/AKT signal transduction pathways in Ishikawa and HEC-1B cells.** (**A** and **B**) Western blot was used to determine the phosphorylation of MAPK pathway node proteins and PI3K/AKT pathway node proteins in Ishikawa and HEC-1B cell lines before and after TMEFF1 transfection. Data are presented as the mean ± SEM (n = 3 per group).*P < 0.05, **P < 0.01, and ***P < 0.001.

To further explore the mechanism through which TMEFF1 affects the malignant behavior of endometrial carcinoma cells, we examined the pathway node proteins and found that, compared with the control group, knockdown of the TMEFF1 gene decreased the p-MEK/MEK, p-ERK/ERK, p-PI3K/PI3K, and p-AKT/AKT ratios (Fig. 8A and B). These results indicate that TMEFF1 activates the MAPK and PI3K/AKT signaling pathways in endometrial carcinoma.

### The transcription factor p53 in endometrial cancer cell lines cannot directly regulate the expression of TMEFF1

**Figure 9:**
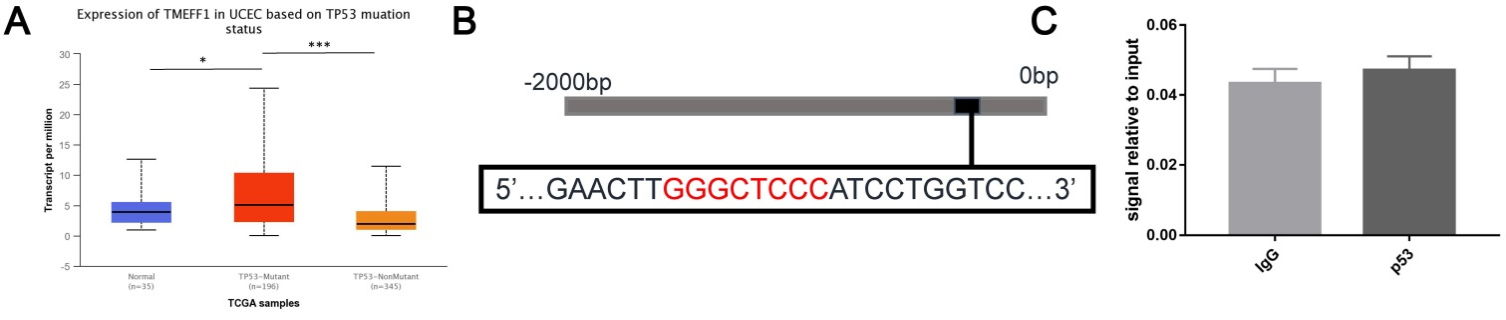
** TMEFF1 is not regulated by p53 in endometrial carcinoma cells.** (**A**) Relative expression of TMEFF1 in normal individuals and UCEC patients with different TP53 mutation statuses. (**B**)Predicted p53 binding sites in the promoter region of TMEFF1. (**C**) The promoter region of TMEFF1 did not bind with the transcription factor p53 in Ishikawa cells. Data are presented as the mean ± SEM (n = 3 per group).

TP53 gene mutations are more abundant in endometrial cancer tissue than normal endometrial tissue. TP53 mutations are closely related to tumors. Further analysis using the UALCAN website found that the expression level of TMEFF1 mRNA in the TP53-mutant group was higher than that in the normal control group and TP53-unmutated group (*P* < 0.05, *P* < 0.001) (Fig. 9A), indicates that TMEFF1 may play an important role in UCEC with TP53 mutations. Further exploration of the regulatory effect of the transcription factor p53 on TMEFF1 was carried out via ChIP experiments, but it was found that p53 cannot bind to the promoter region of TMEFF1 in endometrial cancer cells (*P*>0.05) (Fig. 9B and C).

## Discussion

The vast majority of endometrial carcinoma occurs in postmenopausal women. However, the age of incidence is becoming younger. About 25% of endometrial carcinoma cases are advanced when diagnosed. These patients have an unsatisfactory objective response rate to chemotherapy and endocrine therapy and poor prognosis. However, the molecular mechanisms of endometrial carcinoma development, progression, and invasion/migration have not been clearly identified 20-22. The *TMEFF1* gene was originally discovered as a gene encoding the secretory protein of the pituitary gland of *Xenopus laevis*, called X7365, also known as tomoregulin-1 23. Early research into TMEFF1 focused on regulation of the central nervous system and embryonic development 24. TMEFF1 was found to be associated with neurological diseases, including Parkinson's disease and GM2 gangliosidosis 25, 26. Subsequently, TMEFF1 was confirmed to be a member of the CTA family 7. The CTA family is involved in the occurrence and development of cancers and is currently a research topic of interest in cancer immunodiagnosis and immunotherapy 4, 5. The expression of the CTA family XAGE-1b is significantly higher in liver cancer tissues and peripheral blood than in a non-liver cancer group. Because the expression level of XAGE-1b is closely associated with the 1-year recurrence rate of patients 27, it can be useful for early diagnosis and prognosis. The expression levels of the CTA family members MAGE-3 and MAGE-4 mRNA are significantly higher in the peripheral blood of metastatic hepatocellular carcinoma patients than in a non-metastasis group, and they can be used to determine whether hepatoma cells undergo blood vessel metastasis 28.

As a new member of the CTA family, the function of TMEFF1 is not yet clear. In this study, we sought to explore the role of TMEFF1 in endometrial carcinoma and its mechanisms. We found that TMEFF1 is highly expressed in endometrial carcinoma tissues and promotes the invasion and migration of endometrial carcinoma cells. However, it is worth noting that the early research into TMEFF1 in cancer indicated that it acts as a cancer suppressor, highly expressed in normal human brain tissues and lowly expressed in brain malignant cancers. In addition, in four kinds of brain cancer cells, TMEFF1 exhibited medium and low expression, with expression of TMEFF1 in brain cancers causing growth inhibition 9. However, high expression of TMEFF1 was also detected in various cancer cell lines such as prostate cancer, ovarian cancer, and pancreatic cancer, but its function is not known 8, 29.

Through the Oncomine database and GEPIA online analysis tool TMEFF1 was found to be significantly highly expressed in esophageal cancer, gastric cancer, ovarian cancer, pancreatic cancer, prostate cancer and uterine corpus endometrial carcinoma. To further explore the relationship between the expression of TMEFF1 in endometrial cancer and the prognosis, Kaplan-Meier survival analysis via UALCAN showed that the overall survival (OS) of patients with high expression of TMEFF1 was significantly worse than that of patients with low expression, indicating that high expression of TMEFF1 can indicate a poor prognosis of patients with endometrial cancer and that TMEFF1 expression has potential as a molecular marker for clinical diagnosis and prognostic evaluation.

Previous studies have found that TMEFF1 is highly expressed in ovarian cancer and is an independent risk factor for prognosis. TMEFF1 promotes the malignant behavior of ovarian cancer by activating the PI3K/AKT and MAPK pathways 10. TMEFF1 plays a distinctly different role in cancerogenesis in different cancer tissues. To further verify the expression of TMEFF1 in endometrial cancer tissues and analyze its relationship with clinicopathological parameters and prognosis, in this study, the expression of TMEFF1 was determined in 135 endometrial tissue samples by immunohistochemistry. Our results showed that TMEFF1 was highly expressed in endometrial carcinoma, that its expression was closely associated with FIGO stage and lymph node metastasis, and that it was an independent predictor of survival. The results also showed that the high expression rate of TMEFF1 in the atypical endometrial hyperplasia group was significantly higher than that in the normal endometrium group, and lower than that in the malignant group, which demonstrates that the expression of TMEFF1 was related to the malignant progression of endometrial cancer, and can monitor the malignant transformation and prognosis in patients with atypical endometrial hyperplasia. The above data demonstrate that TMEFF1 has malignant potential in human endometrial carcinoma.

More than 90% of solid cancer deaths are largely due to metastasis 30. Cancer metastasis is a complex process that includes invasion, neovascularization and intravascular penetration, survival and anti-anoikis in the blood circulation, and extravasation and colonization 30-33. However, the mechanisms of the above process remain unclear. In the case of independent expression, TMEFF1 was preferentially expressed in the cell membrane. However, after the binding of TMEFF1 to addicsin, which is a new protein in the amygdala of mice, the distribution of TMEFF1 in the cell membrane was inhibited so that TMEFF1 mainly redistributed to the endoplasmic reticulum and suppressed the migration of cells 34. This indicates that TMEFF1 participates in the migration process of cells. In this study, the results of cell scratch and Transwell assays showed that TMEFF1 knockdown inhibits cell invasion and migration in Ishikawa and HEC-1B cells. These results support the hypothesis that TMEFF1 is a cancer-promoting gene in endometrial carcinoma. The EMT process is considered to be a key mechanism of metastatic changes. Many CTA family members (e.g., SSX, MAGE-D4B, CAGE, piwil2, and CT45A1) are involved in the regulation of cancer cell EMT, upregulating EMT and metastatic genes and promoting EMT and cancer metastasis 35. We found that TMEFF1 inhibited the expression of E-cadherin and upregulated the mesenchymal marker vimentin and the metalloproteinases MMP2 and MMP9. These results indicate that TMEFF1 is involved in promoting EMT. Studies have shown that when the EMT transcriptional factors Snail, Slug, and E47 are upregulated, TMEFF1 expression is elevated 36. These transcription factors are significant inducers of EMT and strongly inhibit the expression of E-cadherin 36, 37, indicating that TMEFF1 is the intermediate link between these transcription factors affecting E-cadherin.

Mutation in the tumor suppressor gene TP53 is associated with aggressive and advanced tumors 38. It is more common in high-grade, poorly differentiated, and progesterone receptor-negative cases of endometrial cancer than in low-risk cases 39. The prognosis of endometrial cancer patients with TP53 mutation is worse than that of patients without mutation 39. We found that in endometrial cancer, the expression level of TMEFF1 mRNA in the TP53-mutant group was higher than that in the normal endometrial group and the TP53-unmutated group. This is consistent with the previous discovery that TMEFF1 is highly expressed in TP53-mutant breast cancer 40, indicating that TMEFF1 plays a greater role in TP53-mutant tumors. We previously confirmed that the expression of TMEFF1 in ovarian cancer is regulated by the transcription factor p53 (mutant)^10^, but the same regulatory effect has not been found in endometrial cancer, indicating that there may be other links between TMEFF1 and TP53 in endometrial cancer, which need to be further explored.

Through the cBioPortal database, it was found that TMEFF1 is genetically altered in endometrial cancer, with alterations mainly including amplifications, deep deletions, truncating mutations and missense mutations. However, these mutations had no significant effect on OS, DFS, PFS or DSS in patients with endometrial cancer. To better clarify the molecular mechanism, we further constructed a gene co-expression network with TMEFF1 and their top 50 neighbor genes, suggesting that TMEFF1 may exert their function by interacting with proteins. Studies have found that the TMEFF1 domain EGF can interact with the CUB domain of ST14, and such an interaction activates ST14 29, which play a role in the degradation of extracellular matrix and the promotion of tumor growth, infiltration, metastasis and other malignant processes 41. This indicates that TMEFF1 can participate in tumor progression through its interacting proteins. GO enrichment analysis showed that TMEFF1 and its related genes are mainly located in the transferase complex, nuclear body, chromosomal region and other regions, combined with chromatin, transcription factor, histone, etc., and can regulate the activity of DNA-dependent ATPase, transcription coregulator, ubiquitin-like protein transferase and other enzymes, indicating that TMEFF1 and its related genes are mainly involved in gene epigenetics and cell biological signal transduction. Many studies have shown that TMEFF1 can regulate multiple molecular mechanisms and related signal pathways. TMEFF1 prevents the binding of Cripto-1 to the ALK4 receptor by binding to the CFC domain of Cripto-1, thereby inhibiting nodal signaling 42, 43; TMEFF1 acts as a downstream signaling factor of TGF-βR2 and acts directly on Smad2/3, inhibiting the BMP signal to reduce the threshold required for the growth inhibitory signal of hair follicle stem cells, thereby promoting hair follicle stem cell self-renewal 44. TMEFF1 regulates the development of cardiac hypertrophy in TAC-induced cardiac hypertrophy by inhibiting TGFβ noncanonical (TAK1-JNK) pathways in the myocardium 45.

KEGG enrichment analysis revealed significant enrichment in the cell cycle, Hippo signaling, spliceosome, RNA transport and other pathways. The above signaling pathways can participate in the occurrence and development of a variety of tumors and are closely related to the occurrence and development of UCEC 46-49. This study found that TMEFF1 promotes the malignant behavior of endometrial carcinoma cells by activating the MAPK and PI3K/AKT signaling pathways, which is consistent with previous findings in ovarian cancer, suggesting that this generality may be related to the EGF-like domain of TMEFF1 10. TMEFF1 is a member of the EGF family because its extracellular region contains a modified EGF-like domain that is highly homologous to the growth factors of the EGF family. The EGF family is closely associated with the occurrence and development of cancers. When EGFR binds to the ligands, the receptors autophosphorylate and transmit information along various downstream signaling pathways such as PI3K/AKT and MAPK to promote cell proliferation and increase cancer cell migration and invasion 50, 51. The EGF-like domain of TMEFF1 can phosphorylate tyrosine of the erbB4 receptor 23, 24, 52, which in turn may activate the MAPK and PI3K/AKT pathways to promote the malignant behavior of cancers.

Targeted drugs for inhibiting the MAPK or PI3K pathway have been used clinically but, because the targeting specificity is not precise enough, the resulting adverse effects are a concern and more specific drug targets urgently need to be developed 53-56. The CTA NY-ESO-1, a homologous family member of TMEFF1, can induce a higher antibody titer and CD4^+^/CD8^+^ T cell immune response, with a vaccine treatment prolonging the progression-free survival of patients with advanced ovarian cancer. It will thus be possible to develop monoclonal immunosuppressive agents that induce specific immune responses 57. As an upstream factor of the MAPK or PI3K pathway, the high expression and carcinogenesis of TMEFF1 in endometrial carcinoma may have potential for the diagnosis of diseases, monitoring of cancer progression, and immunotargeted therapy, and become a new target for the treatment of endometrial carcinoma.

## Conclusions

Based on the results of the current study, we confirmed that TMEFF1 plays an important role in endometrial carcinoma. As an independent prognostic factor for endometrial carcinoma, TMEFF1 promotes the invasion and migration of endometrial carcinoma cells, activates the PI3K/AKT and MAPK signaling pathways, and participates in the regulation of EMT. It may be useful as a prognostic biomarker and therapeutic target.

## Supplementary Material

Supplementary tables.

Supplementary table S7.

## Figures and Tables

**Table 1:** Expression of TMEFF1 in different endometrial tissues

Groups	Cases	Low		High		Positive rate (%)	High expression rate (%)
		(-)	(+)	(++)	(+++)		
Malignant	75	13	19	27	16	82.67^ab^	57.33^c^
Atypical	24	9	4	11	0	62.50	45.83
Severe	7	1	2	4	0	85.71	57.14
Moderate	9	4	0	5	0	55.56	55.56
Mild	8	4	2	2	0	50.00	25.00
Normal	36	15	12	8	1	58.33	25.00
Proliferative	15	7	5	3	0	53.33	20.00
Secretory	21	8	7	5	1	61.90	28.57

Note: The positive rate of TMEFF1 was significantly higher in the malignant group than in the atypical and normal groups (*P^a^*=0.039, *P^b^*=0.006). The high expression rate of TMEFF1 was significantly higher in the malignant group than in the normal group (*P^c^*=0.001).

**Table 2:** Relationship between TMEFF1 and clinical pathological parameters of endometrial carcinoma

Characteristics	Cases	Low		High		Positive rate (%)	*P*	High expression rate (%)	*P*
		(-)	(+)	(++)	(+++)				
**Age at diagnosis, years**									
<59	37	5	13	15	4	86.49	*P*>0.05	51.35	P>0.05
≥59	38	8	6	12	12	78.95		63.16	
**FIGO stage**									
I	50	12	13	18	7	76.00	*P*=0.021	50.00	*P*=0.027
II	6	1	2	2	1	83.33		50.00	
III	16	0	3	6	7	100.00		81.25	
IV	3	0	1	1	1	100.00		66.67	
**Pathologic type**									
Endometrioid	37	5	9	16	7	86.49	*P*>0.05	62.16	*P*>0.05
Serous	23	5	6	7	5	78.26		52.17	
Clear cell	8	1	2	2	3	87.50		62.50	
Others	7	2	2	2	1	71.43		42.86	
**Differentiation**									
Well	16	2	3	8	3	87.50	*P*>0.05	68.75	*P*>0.05
Moderate	29	6	10	8	5	79.31		44.83	
Poor	30	5	6	11	8	83.33		63.33	
**ER**									
-	39	6	11	10	12	84.62	*P*>0.05	56.41	*P*>0.05
+	27	5	7	14	1	81.48		55.56	
Unknown	9	2	1	3	3	77.78		66.67	
**PR^b^**									
-	38	5	11	13	9	86.84	*P*>0.05	57.89	*P*>0.05
+	28	6	7	11	4	78.57		53.57	
Unknown	9	2	1	3	3	77.78		66.67	
**Muscular invasion**									
<1/2	48	9	13	17	9	81.25	*P*>0.05	54.17	*P*>0.05
≥1/2	27	4	6	10	7	85.19		62.96	
**LN metastasis^c^**									
-	48	13	9	17	9	72.92	*P*=0.029	54.17	*P*>0.05
+	14	0	4	5	5	100.00		71.43	
Unknown	13	0	6	5	2	100.00		53.85	

a: Nine patients without ER detection. b: Nine patients without PR detection. c: Thirteen patients without lymphadenectomy.

**Table 3:** Univariate Kaplan-Meier prognosis analysis of endometrial carcinoma

Variable	Characteristics	(Log-rank) *P*-value
Age at diagnosis	< 59 years vs ≥59 years	0.011
FIGO stage	I-II vs III-IV	<0.001
Differentiation grade	Well-moderate vs poor	0.026
Muscular invasion	< 1/2 vs ≥1/2	0.001
LN metastasis	Negative vs positive	<0.001
PR	Negative vs positive	<0.001
ER	Negative vs positive	0.009
TMEFF1	Low vs high	0.035

**Table 4:** Univariate and multivariate Cox regression analysis of patients with endometrial carcinoma

Variables	Univariate analysis		Multivariate analysis	
	*P*-value	Hazard ratio (95%CI)	*P*-value	Hazard ratio (95%CI)
Age at diagnosis	0.017	2.743 (1.199-6.273)	0.929	0.942 (0.253-3.503)
FIGO stage	<0.001	7.640 (3.488-16.732)	0.004	27.107 (2.795-262.904)
Differentiation grade	0.033	2.281 (1.067-4.877)	0.323	1.748 (0.578-5.288)
Muscular invasion	0.002	3.276 (1.528-7.025)	0.025	4.948 (1.223-20.026)
LN metastasis	<0.001	5.183 (2.255-11.911)	0.213	0.234 (0.024-2.308)
PR	0.002	0.104 (0.024-0.445)	0.314	0.314 (0.043-2.285)
ER	0.016	0.262 (0.088-0.776)	0.18	0.398 (0.103-1.533)
TMEFF1	0.044	2.421 (1.023-5.728)	0.302	3.037 (0.368-25.068)

**Table 5:** The Kinase, miRNA and transcription factor-target networks of TMEFF1 in Uterine Corpus Endometrial Carcinoma

Enriched Category	Geneset	LeadingEdgeNum	FDR
Kinase Target	ATM serine/threonine kinase	63	0.002129
	TTK protein kinase	7	0.055353
	ATR serine/threonine kinase	13	0.055353
	polo like kinase1	11	0.0599611
	cyclin dependent kinase 2	3	0.18948
miRNA Target	CTTGTAT,MIR-381	110	0
	GTATGAT,MIR-154,MIR-487	37	0
	ACTGAAA,MIR-30A-3P,MIR-30E-3P	89	0
	GTATTAT,MIR-369-3P	110	0
	GACTGTT,MIR-212,MIR-132	68	0
Transcription Factor Target	V$E2F1_Q6	94	0
	V$E2F_02	93	0
	V$E2F1DP1_01	92	0
	V$E2F1DP2_01	92	0
	V$E2F4DP2_01	92	0

## Abbreviations

UCEC: uterine corpus endometrial carcinoma
ChIP: chromatin immunoprecipitation assay
GO: Gene Ontology
KEGG: Kyoto Encyclopedia of Genes and Genomes
EMT: epithelial-to-mesenchymal
CTAs: Cancer/testis antigens
FIGO: International Federation of Gynecology and Obstetrics
ER: estrogen receptor
PR: progesterone receptor
LN: lymph node
SDS-PAGE: sodium dodecyl sulfate-polyacrylamide gel electrophoresis
