# Overexpression of TMEFF1 in Endometrial Carcinoma and the Mechanism Underlying its Promotion of Malignant Behavior in Cancer Cells: Erratum

**DOI:** 10.7150/jca.113036

**Published:** 2025-03-28

**Authors:** Xin Nie, Lingling Gao, Mingjun Zheng, Caixia Wang, Shuang Wang, Xiao Li, Yue Qi, Liancheng Zhu, Juanjuan Liu, Bei Lin

**Affiliations:** 1 Shengjing Hospital of China Medical University, Department of Obstetrics and Gynecology, Shenyang, China.; 2 Key Laboratory of Maternal-Fetal Medicine of Liaoning Province, Key Laboratory of Obstetrics and Gynecology of Higher Education of Liaoning Province, Shenyang, China.; 3 University Hospital, LMU Munich, Department of Obstetrics and Gynecology, Munich, Germany.; 4 West China Second University Hospital, Sichuan University, Department of Obstetrics and Gynecology, Sichuan, China.

In the original version of our article, there was an error in Fig. 7A. Specifically, western blot band for E-cadherin protein of HEC-IB cells in Figure 7A is incorrect. The correct image is provided below. This correction will not affect the results and conclusions. We apologize for any inconvenience this may have caused.

## Figures and Tables

**Figure 7:**
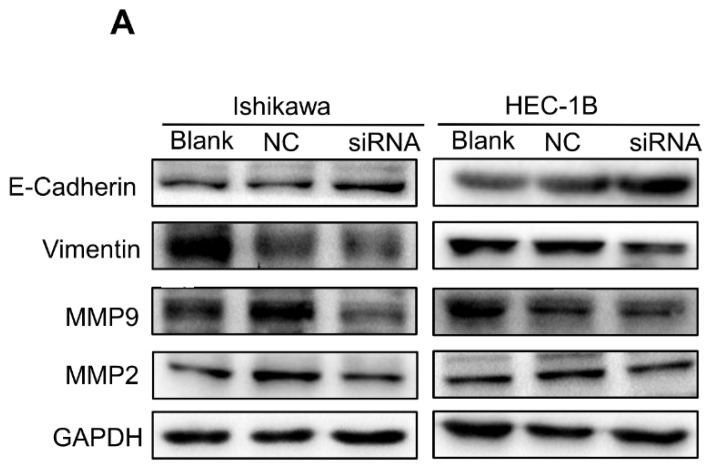
TMEFF1 is involved in the promotion of epithelial-mesenchymal transition in Ishikawa and HEC-1B cells. (A and B) Western blot analysis indicating that TMEFF1 increases the expression of vimentin, MMP2, and MMP9 in Ishikawa and HEC-1B cells and decreases the expression of E-cadherin. Data are presented as the mean ± SEM (n = 3 per group). *P < 0.05, **P < 0.01, and ***P < 0.001.

