# Supplementary material for: Overexpression of TMEFF1 in Endometrial Carcinoma and the Mechanism Underlying its Promotion of Malignant Behavior in Cancer Cells
Source: J Cancer. 2021 Jul 31;12(19):5772–88. doi: 10.7150/jca.58524 (PMC8408118; doi:10.7150/jca.58524)
Supplement: Supplementary file 1 — Supplementary tables. [file jcav12p5772s1.pdf]

Supplementary tables

Supplementary table 1. Significantly enriched GO annotations (Cellular Components) of TMEFF1 in endometrial carcinoma in Metascape

GO	Category	Description	Count	%	Log(P)	$Log_{10}(q)$
GO:1990234	GO Cellular Components	transferase complex	55	11.11	-15.22	-11.93
GO:0016604	GO Cellular Components	nuclear body	53	10.71	-13.52	-10.53
GO:0098687	GO Cellular Components	chromosomal region	30	6.06	-10.34	-7.53
GO:0005819	GO Cellular Components	spindle	29	5.86	-9.5	-6.81
GO:0031965	GO Cellular Components	nuclear membrane	26	5.25	-9.2	-6.69
GO:0034708	GO Cellular Components	methyltransferase complex	13	2.63	-6.66	-4.28
GO:0005681	GO Cellular Components	spliceosomal complex	17	3.43	-6.39	-4.16
GO:0034399	GO Cellular Components	nuclear periphery	14	2.83	-6.36	-4.16
GO:0005667	GO Cellular Components	transcription factor complex	23	4.65	-5.82	-3.76
GO:0000123	GO Cellular Components	histone acetyltransferase complex	10	2.02	-5.01	-3.07
GO:0045120	GO Cellular Components	pronucleus	5	1.01	-4.91	-3.02
GO:0016592	GO Cellular Components	mediator complex	6	1.21	-4.1	-2.36
GO:0018995	GO Cellular Components	host	8	1.62	-3.89	-2.2
GO:0042382	GO Cellular Components	paraspeckles	3	0.61	-3.8	-2.15
GO:0000792	GO Cellular Components	heterochromatin	8	1.62	-3.69	-2.1
GO:0016605	GO Cellular Components	PML body	8	1.62	-2.97	-1.45
GO:0030496	GO Cellular Components	midbody	11	2.22	-2.91	-1.39
GO:0071564	GO Cellular Components	npBAF complex	3	0.61	-2.8	-1.29
GO:0015030	GO Cellular Components	Cajal body	6	1.21	-2.8	-1.29
GO:0000781	GO Cellular Components	chromosome, telomeric region	10	2.02	-2.76	-1.27

Supplementary table 2. Significantly enriched GO annotations (Biological Processes) of TMEFF1 in endometrial carcinoma in Metascape

GO	Category	Description	Count	%	Log(P)	$Log_{10}(q)$
GO:0006397	GO Biological Processes	mRNA processing	47	9.49	-17.36	-13.16
GO:0018205	GO Biological Processes	peptidyl-lysine modification	32	6.46	-10.34	-6.92
GO:0006403	GO Biological Processes	RNA localization	22	4.44	-8.46	-5.17
GO:0051301	GO Biological Processes	cell division	37	7.47	-8.42	-5.17

GO:0010564	GO Biological Processes	regulation of cell cycle process	43	8.69	-8.18	-5.02
GO:0006338	GO Biological Processes	chromatin remodeling	18	3.64	-7.32	-4.26
GO:0006310	GO Biological Processes	DNA recombination	23	4.65	-7.27	-4.24
GO:0006275	GO Biological Processes	regulation of DNA replication	13	2.63	-6.47	-3.65
GO:0051304	GO Biological Processes	chromosome separation	12	2.42	-6.44	-3.64
GO:0003002	GO Biological Processes	regionalization	23	4.65	-5.94	-3.21
GO:0043414	GO Biological Processes	macromolecule methylation	21	4.24	-5.81	-3.11
GO:0001654	GO Biological Processes	eye development	23	4.65	-5.8	-3.11
GO:0006352	GO Biological Processes	DNA-templated transcription, initiation	18	3.64	-5.32	-2.8
GO:1990090	GO Biological Processes	cellular response to nerve growth factor stimulus	8	1.62	-5.23	-2.74
GO:0019827	GO Biological Processes	stem cell population maintenance	14	2.83	-5.22	-2.74
GO:0072331	GO Biological Processes	signal transduction by p53 class mediator	18	3.64	-4.97	-2.56
GO:0043632	GO Biological Processes	modification-dependent macromolecule catabolic process	31	6.26	-4.96	-2.55
GO:0046831	GO Biological Processes	regulation of RNA export from nucleus	5	1.01	-4.91	-2.51
GO:0080135	GO Biological Processes	regulation of cellular response to stress	34	6.87	-4.72	-2.4
GO:0007420	GO Biological Processes	brain development	33	6.67	-4.61	-2.33

Supplementary table 3. Significantly enriched GO annotations (Molecular Functions) of TMEFF1 in endometrial carcinoma in Metascape

GO	Category	Description	Count	%	Log(*P*)	$Log_{10}(q)$
GO:0003682	GO Molecular Functions	chromatin binding	45	9.09	-14.49	-10.82
GO:0008094	GO Molecular Functions	DNA-dependent ATPase activity	11	2.22	-6.86	-3.5
GO:0008134	GO Molecular Functions	transcription factor binding	35	7.07	-6.56	-3.38

GO:0003712	GO Molecular Functions	transcription coregulator activity	32	6.46	-6.25	-3.18
GO:0042393	GO Molecular Functions	histone binding	17	3.43	-6.05	-3.08
GO:0019904	GO Molecular Functions	protein domain specific binding	34	6.87	-5.37	-2.48
GO:0031490	GO Molecular Functions	chromatin DNA binding	11	2.22	-4.89	-2.13
GO:0017056	GO Molecular Functions	structural constituent of nuclear pore	6	1.21	-4.76	-2.04
GO:1990226	GO Molecular Functions	histone methyltransferase binding	4	0.81	-4.71	-2.04
GO:0019900	GO Molecular Functions	kinase binding	33	6.67	-4.61	-1.99
GO:0019787	GO Molecular Functions	ubiquitin-like protein transferase activity	22	4.44	-4.42	-1.9
GO:0003697	GO Molecular Functions	single-stranded DNA binding	10	2.02	-3.88	-1.49
GO:0008022	GO Molecular Functions	protein C-terminus binding	13	2.63	-3.84	-1.49
GO:0046332	GO Molecular Functions	SMAD binding	8	1.62	-3.81	-1.49
GO:0015631	GO Molecular Functions	tubulin binding	18	3.64	-3.53	-1.25
GO:0004674	GO Molecular Functions	protein serine/threonine kinase activity	21	4.24	-3.45	-1.2
GO:0003729	GO Molecular Functions	mRNA binding	23	4.65	-3.33	-1.13
GO:0000287	GO Molecular Functions	magnesium ion binding	13	2.63	-3.31	-1.13
GO:0042826	GO Molecular Functions	histone deacetylase binding	9	1.82	-3.3	-1.13
GO:0001098	GO Molecular Functions	basal transcription machinery binding	7	1.41	-3.26	-1.13

Supplementary table 4. Significantly enriched KEGG pathway annotations of TMEFF1 in endometrial carcinoma in Metascape

GO	Category	Description	Count	%	Log(*P*)	$\text{Log}_{10}(q)$
hsa04110	KEGG Pathway	Cell cycle	12	2.42	-5.06	-2.5
hsa04390	KEGG Pathway	Hippo signaling pathway	13	2.63	-4.78	-2.5
hsa03040	KEGG Pathway	Spliceosome	12	2.42	-4.71	-2.5
hsa03013	KEGG Pathway	RNA transport	13	2.63	-4.3	-2.21
hsa05220	KEGG Pathway	Chronic myeloid leukemia	8	1.62	-4.02	-2.03
hsa04360	KEGG Pathway	Axon guidance	11	2.22	-3.03	-1.24
hsa04330	KEGG Pathway	Notch signaling pathway	5	1.01	-2.55	-0.93
hsa04520	KEGG Pathway	Adherens junction	6	1.21	-2.46	-0.88

hsa05166	KEGG Pathway	HTLV-I infection	12	2.42	-2.19	-0.67
hsa04310	KEGG Pathway	Wnt signaling pathway	8	1.62	-2.05	-0.61
hsa03440	KEGG Pathway	Homologous recombination	4	0.81	-2.03	-0.61

Supplementary table 5. Significantly enriched kinase-target networks of TMEFF1 in endometrial carcinoma in LinkedOmics

GeneSet	*P*-Value	FDR	LeadibfEdgeGene
Kinase_ATM	0	0	CREB1 TOPBP1 RIF1 ZNF420 SMC3 USP34 DYRK2 SMAD1 TERF1 DCK RANBP9 APLF ATF2 PRKDC RNF138 ZBTB2 BUB1 PBRM1 MCM3 ZNF148 HMGA2 SIAH1 ATF1 RAD18 UBR5 RAD17 CCDC6 RBBP8 ITCH DCLRE1A ABL1 BLM RAD50 CHD4 MED1 SMC1A RNF20 HUWE1 STRAP EZH2 ATM WRN NUSAP1 MDC1 RFWD3 EP300 FUS HIPK2 DBF4 RPA1 PPP1R2 FANCD2 NBN TP63 EYA3 TDP1 EXO1 USP10 RRM2B CHEK1 USP28 UIMC1 KDM2A
Kinase_ATR	0	0.0428	CREB1 CGGBP1 DCK PRKDC MCM3 SIAH1 IRS1 RAD17 MCM2 CLSPN RBBP8 BLM CCDC82 SMC1A HUWE1 ATM WRN MDC1 DBF4 FANCD2 RAD1 MARCKSL1 NBN ATR PHF14 TDP1 CHEK1 UIMC1
Kinase_TTK	0.005128	0.0436	BUB1B TTK CDCA8 ARHGEF17 ABL1 BLM PRC1
Kinase_PLK1	0	0.051	NINL CENPQ YY1 NEDD1 BRCA2 CDC23 TOP2A FBXO5 TERF1 TOPORS BUB1B SNCA HNRNPUCE P192 ESPL1 IRS1 ANAPC1 ZMYM2 CLSPN RSF1 FOXM1 USP16 CCNT1 RACGAP1 SUZ12 PRC1 CTNNB1 MAP9 ERCC6L CEP55 PTPN1 DVL2 FBXO43 STIL SVIL GTSE1 KIF2A AXIN2 WEE1 CPEB1 CDC27 CCNB1 RUVBL1 BRCA1 CDC25C CDC25A
Kinase_CSNK 1A1	0.002381	0.0564	CREB1 YWHAQ LRP6 SNCA APC GLI3 ATF1 HNRNPC VHL YWHAZ CTNNB1 RAPGEF2 KCNIP3

LeadingEdgeNum: the number of leading edge genes; FDR: false discovery rate from Benjamini and Hochberg from gene set enrichment analysis (GSEA)

Supplementary table 6. Significantly enriched miRNA-target networks of TMEFF1 in endometrial carcinoma in LinkedOmics

GeneSet	*P*-Value	FDR	LeadibfEdgeGene
CTTGTAT,MIR-381	0	0	PAPOLG PSIP1 PRMT6 RNF2 KLF12 CGGBP1 RNF144A KALRN ZNF532 HNRNPR PDS5B ZFAND3 KHDRBS1 STAG1 GTF2I 11-Sep ST8SIA2 RRAGB DHX40 WNT5A ANKRD50 APPBP2 HNRNPH2 PRPF38B MSL2 KIF1B QKI ZMYND11 PHF2 ATRN RHOQ ACVR2A CCNT2 RAB11FIP2 SIAH1 HBP1 MED13 UBR5 B3GNT5 LARP1 SRRM1 EIF4G2 SYNCRIP BIRC6 ROCK2 GFRA1 AZIN1 R3HDM2 JPH1 ZBTB22 SLC38A2 ITCH RPS6KA3 EIF3A KPNA3 EPHA4 PAPOLA DDX6 PURG ARGLU1 CHD4 EIF4G3 KCTD3 UBR3 PAN3 ELOVL7 MED13L FAM117A NBEA CNOT7 RNF13 ZNF770 R3HDM1 RPS6KB1 ANKRD12 MED14 SNRNP40 PDGFC OSBPL3 RAP2C RC3H1 MPHOSPH9 VCPIP1 ZFPM2 SEMA3C GJA1 PARD6B GORASP2 PDS5A GRM1 MECOM UBE2E2 SOX4 ASXL2 EPB41 MBNL2 GAS2 ARID4B RNF111 NAV3 WEE1 LCOR PNRC1

			TBC1D15 ABCA1 SLMAP BAGE C15orf41 BTBD7 BCL11A
GTATGAT,MIR-154,MIR-487	0	0	SP3 YTHDC1 SPRED2 RNF44 BMI1 RNF38 MAGI2 SIRT1 ZMYM4 TEAD1 SMAD7 ARID1A HNRNPH2 TOP1 RBBP6 DYRK1A ZNF281 PUM2 DNMT3A AZIN1 CAPRIN1 KDM6A SNX6 TRIB2 ATP11C KPNA3 FMR1 KIAA1217 ARID2 GALC PPM1A UBE2Q1 SNRNP40 ASAP2 RC3H1 RFX3 SEMA3C
ACTGAAA,MIR-30A-3P,MIR-30E-3P	0	0	SP3 PAPOLG PSIP1 ZNF22 USP1 SAP130 CBL TCF7L1 C5orf30 ZNF280B LMNB1 FXR1 PAIP2 TSC22D2 NR2C2 FAM13B HNRNPH2 RBMX TOP1 QKI KRAS FOXJ3 PBRM1 OSBPL11 APC RBMXL1 TFDP1 HMGA2 C5orf24 SIAH1 ZFYVE9 ELK3 TBL1XR1 TNRC6B ZFX DNAJC27 CSNK1G3 TNPO3 PPP2R5E BIRC6 CAPRIN1 CCDC6 USP6 RAB10 RALA GDF6 ARID4A CCKBR DNAJB4 HIRA NRCAM RBM45 RICTOR AKAP9 LRRTM1 CDC40 NAA25 MLLT10 DDX3X KIAA1324L PATZ1 EP300 DBF4 ROBO1 FNDC5 VCPIP1 RARB EYA3 SEMA3C LHX8 MARK1 CSNK1E SIX4 SCAMP1 LRRTM2 ZFAND5 YPEL5 ARPP19 HERC4 CREBBP DNAJB14 AHCYL2 UBE2G1 SFRP1 PRKAA1 NPY2R VGLL3 BRCA1 CDC37L1
ATGTTAA,MIR-302C	0	0	EPC2 SP4 KLHL24 PPP3R1 MATR3 NUP153 YY1 CGGBP1 USP1 NFYB UBA2 DCAF7 GABPA SIRT1 MYO1B SUV39H2 ZMIZ1 FBXL3 SSX2IP TEAD1 B4GALT5 FXR1 HNRNPH1 PPP1R12A INTS2 HNRNPA2B1 SF3B1 LRCH2 RNF138 SENP6 CAND1 EIF4A2 FBXO21 GPRASP2 CCNT2 ARID1B RNF11 MED13 TNRC6B MAPRE1 PCDH9 B3GNT5 PPP2R5E TTC7B USP33 SAMD8 AZIN1 VEZT IRS2 VEZF1 LCA5 CREBZF GOLPH3 FMR1 DAAM1 CHD6 PHF20L1 SMAD4 PCBP1 UBR3 ACVR1 LUC7L3 KIAA0355 VPS8 C18orf25 RBM39 CDK19 FAM117A DDX3X RNF13 STRN SOX11 MYT1 RAI1 PANK3 PHACTR2 EHD3 GABRA2 KIF4A ANKRD12 PARP1 FAM84B ATP2C1 PPARGC1A WNT3 UBE2V2 VCPIP1 KLHDC10 ZFPM2 LRRTM4 CREM PHF14 PDS5A LATS1 LIFR ASF1A ZNF319 GGNBP2 FGFR2 TMEM33 DACH1 RHEB NRP2 ST6GALNAC3 SLC35A1 WDTC1 BTAF1 TBL1X BCL11A CYFIP2 SPRY1 POU3F2 SBF2 NPY2R SH3D19 PPP5C PCF11 BTBD3

CTACTGT,MIR-199A	0	0	SPAST NINL TAB2 YTHDC1 AKIRIN1 PHF6 HECTD2 CBL SNN KDM3A PDS5B AEBP2 ZNF638 FXR1 PPP1R12A PTX3 CTDSPL2 BZW1 USP11 RNF139 NFE2L2 KIN TOP1 FNBP1L QKI ACVR2A CCNT2 NAA15 CXXC5 TNRC6B TGIF2 PPP2R5E SCUBE3 RLIM DNMT3A KDM6A AFF4 TANC1 WSB1 RAB2B FGF12 TAF5 TMEM161B CDK17 GIGYF2 CHD7 LUC7L3 NOVA1 PLAG1 NAA25 RBM39 GOLGA4 SEMA6C BAZ2B EML4 LRRC4C EHD3 RBL2 ZHX1PHF12 RBM25 ATP1B2 UXS1 SUMO3 ARHGAP21 LIFR DPP10 SECISBP2L ARID4B RNF111 NRP2 SORCS3 FAM193A EDEM3 ITGB8 ABCA1 BTBD7 RREB1 PHLPP2 ARRDC3 KCNMB2 AFAP1 GLT8D2 ZNF654 ARHGAP20 PDIK1L ETV1 MTOR GPM6A SIPA1L3

LeadingEdgeNum: the number of leading edge genes; FDR: false discovery rate from Benjamini and Hochberg from gene set enrichment analysis (GSEA)

Supplementary table 7. Significantly enriched transcription factor-target networks of TMEFF1 in endometrial carcinoma in LinkedOmics

GeneSet	*P*-Value	FDR	LeadibfEdgeGene
V$E2F1_Q6	0	0	MSH2 SP3 ZNF644 E2F3 CDC5L MTF2 YTHDC1 TMEM108 PPM1D TOPBP1 RBBP4 SMC3 TRA2B HNRNPR ZNF362 MYH10 STMN1 BRMS1L ATAD5 FBXO5 CBX3 CASP8AP2 ING3 DCK STAG1 HMGXB4 HNRNPA2B1 MAP3K7 UNG SASS6 CTDSPL2 HNRNPD NIPBL PRKDC PRPF4B SMC6 NCL AND1 PBRM1 MCM3 MCM4 EHBP1 MCM8 SMAD6 STT3B TMPO ZNF687 KBTBD7 ATAD2 APH1A E2F8 PHC1 STK35 CTCF MCM2 HNRNPUL1 SYNCRIP CLSPN APPL1 TRMT6 USP37 RBL1 ARHGAP11A ZNF367 ARID4A PHF13 GEN1 POLA1 HIRA STAG2 CCNT1 SERBP1 DMD TFAP4 NELL2 E2F7 SMC1A ZCCHC8 RASAL2 LUC7L3 ILF3 EZH2 POLR2A DNMT1 EPHB1 ATE1 CACNA1G SSBP3 DNAJC9PRPF38A GPN3 FANCD2 MCM6 PHF5A
V$E2F_02	0	0	MSH2 SP3 ZNF644 E2F3 CDC5L MTF2 YTHDC1 TMEM108 PPM1D TOPBP1 SMC3 TRA2B HNRNPR ZNF362 MYH10 STMN1 BRMS1L ATAD5 FBXO5 CBX3 CASP8AP2 ING3 DCK STAG1 HMGXB4 HNRNPA2B1 MAP3K7 UNG SASS6 CTDSPL2 HNRNPD NIPBL PRKDC PRPF4B SMC6 NCL PPIG CAND1 PBRM1 MCM3 MCM4 EHBP1 MCM8 SMAD6 STT3B PLAGL1 TMPO ZNF687 KBTBD7 ATAD2 APH1A E2F8 PHC1 STK35 BRPF1 MCM2 HNRNPUL1 SYNCRIP CLSPN APPL1 TRMT6 USP37 RBL1 ARHGAP11A PCIF1 ZNF367 ARID4A GEN1 POLA1 HIRA STAG2 CCNT1 DMD TFAP4 NELL2 E2F7 SMC1A ZCCHC8 RASAL2 LUC7L3 ILF3 EZH2 POLR2A DNMT1 EPHB1 DNAJC9 FANCD2 MCM6 PHF5A GPBP1 ZNF503 NUP62 TRIM39

V$E2F_Q4	0	0	MSH2 SP3 ABI2 ZNF644 E2F3 MTF2 YTHDC1 TMEM108 PPM1D TOPBP1 NUP153 SMC3 TRA2B HNRNPR ZNF362 MYH10 STMN1 BRMS1L ATAD5 EPC1 FBXO5 PPP1R8 CASP8AP2 ING3 DCK STAG1 CBX5 HMGXB4 UNG SLC38A1 SASS6 CTDSPL2 HNRNPD NIPBL PRKDC SMC6 NCL CAND1 PBRM1 MCM3 MCM4 EHBP1 MCM8 SMAD6 STT3B TMPO ZNF687 KBTBD7 ATAD2 APH1A E2F8 PHC1 STK35 CTCF ZMYM2 MCM2 HNRNPUL1 CLSPN APPL1 USP49 TRMT6 USP37 RBL1 ARHGAP11A JPH1 ZNF367 ARID4A PHF13 GEN1 POLA1 HIRA STAG2 CCNT1 DMD TFAP4 NELL2 E2F7 SMC1A ZCCHC8 RASAL2 LUC7L3 ILF3 EZH2 POLR2A DNMT1 EPHB1 DNAJC9
V$E2F_Q6	0	0	MSH2 SP3 ZNF644 E2F3 MTF2 YTHDC1 TMEM108 PPM1D TOPBP1 NUP153 SMC3 TRA2B HNRNPR ZNF362 MYH10 STMN1 BRMS1L ATAD5 FBXO5 PPP1R8 CASP8AP2 ING3 DCK STAG1 CBX5 HMGXB4 UNG SLC38A1 SASS6 CTDSPL2 HNRNPD NIPBL PRKDC PRPF4B SMC6 NCL CAND1 PBRM1 MCM3 MCM4 EHBP1 MCM8 SMAD6 STT3B TMPO ZNF687 KBTBD7 ATAD2 APH1A E2F8 PHC1 STK35 CTCF ZMYM2 MCM2 HNRNPUL1 CLSPN APPL1 USP49 TRMT6 USP37 RBL1 ARHGAP11A JPH1 ZNF367 ARID4A PHF13 GEN1 POLA1 HIRA STAG2 CCNT1 DMD TFAP4 NELL2 E2F7 SMC1A ZCCHC8 RASAL2 LUC7L3 ILF3 EZH2 POLR2A DNMT1 EPHB1 DNAJC9
V$E2F1DP1_01	0	0	MSH2 SP3 ZNF644 E2F3 CDC5L MTF2 YTHDC1 TMEM108 PPM1D TOPBP1 SMC3 TRA2B HNRNPR ZNF362 MYH10 STMN1 BRMS1L ATAD5 FBXO5 CBX3 CASP8AP2 ING3 DCK STAG1 HMGXB4 HNRNPA2B1 MAP3K7 UNG SASS6 CTDSPL2 HNRNPD NIPBL PRKDC PRPF4B SMC6 NCL PPIG CAND1 PBRM1 MCM3 MCM4 EHBP1 MCM8 SMAD6 STT3B PLAGL1 TMPO ZNF687 KBTBD7 ATAD2 APH1A E2F8 PHC1 STK35 BRPF1 MCM2 HNRNPUL1 SYNCRIP CLSPN APPL1 TRMT6 USP37 RBL1 ARHGAP11A PCIF1ZNF367 ARID4A GEN1 POLA1 HIRA CCNT1 DMD TFAP4 NELL2 E2F7 SMC1A ZCCHC8 RASAL2 LUC7L3 ILF3 EZH2 POLR2A DNMT1 EPHB1 DNAJC9 FANCD2 MCM6 PHF5A GPBP1 ZNF503 NUP62 TRIM39

LeadingEdgeNum: the number of leading edge genes; FDR: false discovery rate from Benjamini and Hochberg from gene set enrichment analysis (GSEA)
